# The Pattern of Cytokine Production *In Vitro* Induced by Ancient and Modern Beijing *Mycobacterium tuberculosis* Strains

**DOI:** 10.1371/journal.pone.0094296

**Published:** 2014-04-11

**Authors:** Yih-Yuan Chen, Jia-Ru Chang, Wei-Feng Huang, Shu-Ching Hsu, Shu-Chen Kuo, Jun-Ren Sun, Horng-Yunn Dou

**Affiliations:** 1 National Institute of Infectious Diseases and Vaccinology, National Health Research Institutes, Zhunan, Miaoli, Taiwan; 2 Division of Clinical Pathology, Department of Pathology, Tri-Service General Hospital and National Defense Medical Center, Taipei, Taiwan; University of Padova, Medical School, Italy

## Abstract

It is unclear to what extent the host-responses elicited by Beijing versus non-Beijing strains of *Mycobacterium tuberculosis* (MTB) contribute to the predominance of modern Beijing strains in Taiwan and some other Asian countries. The purpose of this study was to compare the expression profiles of virulence-related genes in human monocyte-derived macrophages infected in vitro with Beijing (ancient and modern strains) and non-Beijing strains (EAI strains) of MTB that are epidemic in Taiwan. We found that modern Beijing strains induced lower levels of pro-inflammatory cytokines, whereas EAI strains induced higher levels. Notably, the most prevalent modern Beijing sub-lineage, possessing intact RD150 and RD142 chromosomal regions, induced very low levels of pro-inflammatory cytokines, especially interleukin-1β. Moreover, in an intracellular growth assay, the survival of the same modern Beijing strain in human monocyte-derived macrophages was significantly higher than that of an ancient Beijing strain and an EAI strain. Taken together, these results may explain why modern Beijing strains of MTB predominate in Taiwan.

## Introduction

Tuberculosis (TB) continues to be a major infectious and deadly disease throughout the developing world. It has been estimated that 13,000 new TB cases occur each year in Taiwan, based on statistics of the Center for Disease Control, Department of Health [1]. Notably, both the incidence and mortality rates of TB have decreased steadily since 1950; however, it is still an immense public health problem and remains a serious infectious disease in Taiwan. Routes of transmission of Beijing, EAI, Haarlem, LAM and T lineages of *Mycobacterium tuberculosis* (MTB) have been proposed, in which MTB was spread by different waves of ethnic groups over the past centuries [2]. At present, the most common MTB family in Taiwan is the Beijing lineage [3], [4], followed by EAI strains and then Haarlem strains [4].

MTB epidemics have highlighted the roles of both environmental and biological factors in disease transmission, including population density, community hygiene, BCG vaccination, host immune responses and bacterial genes involved in pathogenesis [2], [5], [6], [7], [8]. Pro-inflammatory cytokine signaling is crucial for the host-pathogen immune response, especially against intracellular pathogens like MTB [9], [10]. Mice lacking interleukin-1β (IL-1β), IL-6, interferon-γ (IFN-γ) and tumor necrosis factor-α (TNF-α) showed increased susceptibility to MTB [7]. Previous studies using human monocyte-derived macrophages (MDM) proved that modern lineages of MTB induce mild inflammatory responses whereas ancient lineages induce more robust responses [11], [12]. These results suggest that a reduced immune response to modern MTB lineages contributes to the more rapid disease progression they cause as well as their higher rates of transmission [11], [12].

Evaluation of the virulence of MTB has generally been assessed by measurement of morbidity and mortality in mouse experimental models, whereas cell-culture models are easier to manage and provide more-rapid results [13]. Understanding what determines the transmissibility and pathogenic potential of infection for particular strains of MTB is important for vaccine development and therapy.

The major purpose of this study was to probe the cytokine expression patterns of MDM induced by the three major epidemic MTB strains in Taiwan. In addition, based on RD genotype determination, the relationship between different RD regions (RD181, RD150 and RD142) and cytokine expression induced by the prevalent MTB strains was also investigated. Ultimately, we seek to discover whether cytokine expression patterns induced by the most prevalent MTB stains correlate with transmission dynamics.

## Materials and Methods

### Ethics statement

This study was approved by the Human Ethics Committee of the National Health Research Institutes, Taiwan (Code: EC1010804-E). Because of the retrospective nature of the study, the routine collection of clinical data in daily practice, and the dislinkage of personal information, the requirement to obtain informed consent from MTB patients was waived by our institutional review board.

### Bacteria strains and culture

*M. tuberculosis* H37Rv and clinical strains used in this study (Table S1) were grown on Middlebrook 7H9 medium (Difco Laboratories, Detroit, MI, USA) supplemented with 0.5% glycerol, 0.05% Tween-80, and 10% albumin-dextrose-catalase (ADC) or on solid 7H11 medium (Difco Laboratories) supplemented with oleic acid-albumin-dextrose-catalase (OADC). OADC is added to 7H10 and 7H11 basic media to enhance the growth of mycobacteria.

### Monocyte purification and maturation to monocyte-derived macrophages (MDM)

Blood cells from two healthy male volunteers were used in independent experiments. The two volunteers gave written informed consent for the use of their blood for scientific purposes. Each experiment was repeated in triplicate, and the mean and standard deviation calculated. Monocytes were prepared from healthy donor blood using Ficoll-Paque Plus (GE Healthcare). Following standard procedures, monocytes were then purified using CD14 micro-beads and MACS separation columns (Miltenyi Biotec). Separated monocytes were resuspended in RPMI-1640 medium (Invitrogen) and allowed to attach to 24-well polystyrene plates (Corning, NY, USA) for 2 h at 37°C (1×10^6^ cells/well). After washing with PBS, monocytes were cultured in RPMI-1640 medium supplemented with 10% heat-inactivated human serum (Hyclone), 50 µg/ml penicillin-streptomycin (Invitrogen), and 40 ng/ml granulocyte-monocyte colony-stimulating factor (GM-CSF; ProSpec-Tany TechnoGene, Ltd, East Brunswick, NJ, USA) at 37°C under a 5% CO2, humidified atmosphere. At day 3 and 6, the culture medium was refreshed and supplemented with 40 ng/ml GM-CSF. At day 7, the culture medium was changed to serum-free medium (Invitrogen) and ready for infection.

### MDM infection and intracellular growth assay

MDM were infected with MTB strains at a multiplicity of infection of 1:1 (bacterium to MDM ratio) for 4 h at 37°C under a 5% CO2, humidified atmosphere. The wells were then washed three times with PBS to eliminate extracellular and non-adhering bacteria. Following the last wash, the PBS was replaced with fresh RPMI-1640 medium at 37°C in 5% CO_2_. At selected time points, the bacterial uptake by MDM was determined by lysing a fraction of the cells with 0.1% saponin and counting 50 µl of diluted cell lysates by plating.

### Cytokine analysis

Culture supernatant from control or infected MDM cells was harvested after 72 h and frozen at −80°C. Sterile-filtered culture supernatants were assayed by using a RayBio human cytokine antibody array C series 1000 (AAH-CYT-1000, RayBiotech) or Bio-Plex Pro human cytokine premixed kit (no M50-00031YV, Bio-Rad) according to the manufacturer's instructions.

### Measurement of MTB doubling time

The doubling time of MTB strains was determined as described by Groll *et al* using the MGIT960 system [14]. The tested clinical strains were freshly subcultured on Lowenstein Jensen medium and incubated at 37°C for 3 weeks. Appropriately diluted bacterial suspensions were added in triplicate to Mycobacteria Growth Indicator Tubes (Becton Dickinson Diagnostic Systems, Sparks, MD, USA) supplemented with 10% MGIT960 SIRE™ Supplement (Becton Dickinson, USA). Growth curves and doubling time were obtained by monitoring the fluorescence and recording the growth units every hour using BD EpiCenter™ software.

## Results

### Induction of cytokine expression by local MTB strains in Taiwan correlates with epidemiological surveillance data

To better understand the relationship between virulence and epidemiological surveillance data, we first classified our sampled Beijing strains (the most prevalent lineage in Taiwan), collected during 2003 to 2007 from general hospitals of Taiwan [15], into ancient or modern lineages according to the presence or absence of RD regions (Table 1). Presence of an intact RD181 region identifies the strain as an ancient lineage, whereas absence of RD181 indicates a modern lineage. Out of 338 Beijing strains analyzed, the proportion of modern lineages (90.8%) was significantly higher than that of ancient lineages (9.2%) (Table 1). Furthermore, all isolates were also categorized by MIRU-VNTR typing and cluster analysis (Table S2). The observed high clustering rates indicate that high transmission of modern Beijing strains occurred in Taiwan.

**Table 1:** RD typing of Beijing lineages of *Mycobacterium tuberculosis*.

Beijing sublineages*	No. strains (%)
RD181▪RD150▪ RD142▪	31(9.2)
RD181□RD150 □RD142□	88(26)
RD181□RD150▪ RD142▪	162(47.9)
RD181□RD150▪ RD142□	26(7.7)
RD181□RD150□ RD142▪	31(9.2)
Ancient	31(9.2)
Modern	307(90.8)
Total	338(100)

*The proportions of ancient and modern Beijing strains of *Mycobacterium tuberculosis* were calculated by determining the presence of RD regions.

**Figure 1:**
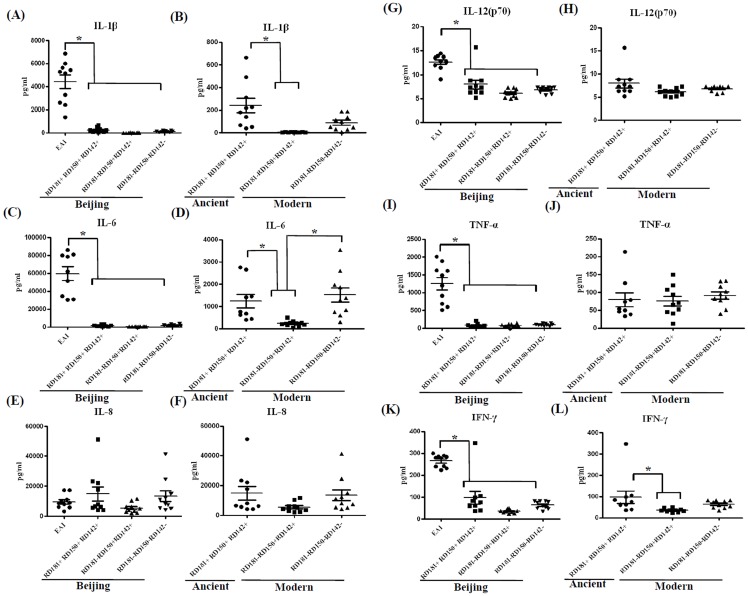
Pro-inflammatory cytokine production varies with genetic clustering of MTB lineages. Ten strains of each sub-lineage were selected for further assays. Human MDM were infected with each MTB strain, respectively. The culture supernatants were collected and assayed as described in methods. *, indicating P<0.05.

In addition, to elucidate relationships between epidemiological surveillance data and cytokine expression, we also investigated EAI strains (the second prevalent lineage in Taiwan) and examined their capacity to induce cytokine expression versus Beijing strains. Comparing the levels of cytokine induction across these three major groups (EAI, ancient and modern Beijing strains) showed that EAI strains consistently induced higher levels of pro-inflammatory cytokines, including IL-1β, IL-6, IL-12, TNF-α and IFN-γ (Fig. 1). Moreover, comparing the levels of cytokine expression between ancient and modern Beijing lineages revealed that the ancient Beijing strains elicited significantly higher levels of IL-1β, IL-6, and IFN-γ (Fig. 1). Taken together, these results show that, following infection of human macrophages, the most prevalent strains, the modern Beijing strains, induce lower levels of pro-inflammatory cytokines, whereas the EAI strains as a group induce higher levels of cytokines.

### Modern Beijing sub-lineages possessing intact RD150 and RD142 regions induce very low levels of pro-inflammatory cytokines

In an attempt to understand the reasons for the differences in cytokine expression patterns between the ancient and modern Beijing lineages, each Beijing sub-lineage was categorized according to the presence of RD regions. The modern Beijing lineage can be further divided into four groups, according to the presence or absence of RD150 or RD142 (Table 1). Two major modern Beijing sub-lineages were chosen for further study (Table 1).

The modern Beijing sub-lineage possessing intact RD150 and RD142 regions induced only very low levels of pro-inflammatory cytokines when compared with the ancient Beijing lineage containing RD181, RD150 and RD142 (Fig. 1). However, the modern Beijing group lacking all three regions (RD181, RD150 and RD142) induced similar pro-inflammatory cytokine levels to the ancient group (containing RD181, RD150 and RD142). The results suggest that a factor (or factors) in the modern Beijing sub-lineage possessing intact RD150 and RD142 regions may function as positive regulator of the immune response.

### Ancient and modern Beijing lineages differ in their induction of pro-inflammatory cytokines

**Figure 2:**
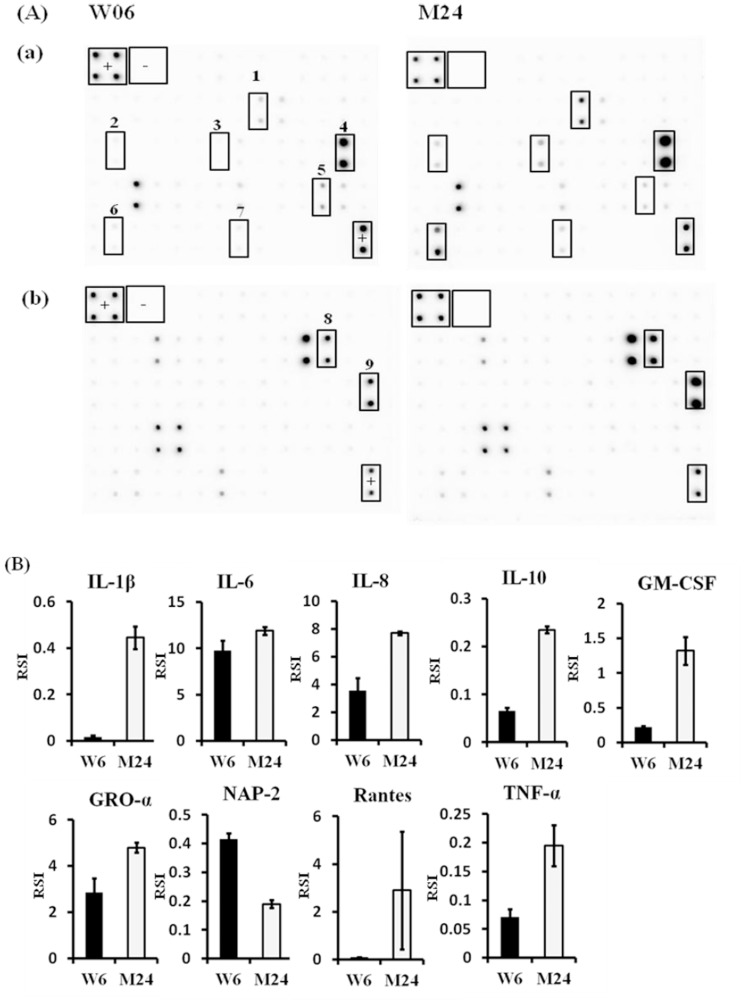
Cytokine arrays reveal differences in cytokine/chemokine expression patterns of MDM infected with *M. tuberculosis* W06 or M24 strains. Human MDM were infected with *M. tuberculosis* W06 or M24 Beijing strains for 72 h, after which the cell culture supernatants were collected and analyzed for cytokine/chemokine content using a RayBio human cytokine antibody array C series 1000 (a and b). The detection signals were captured with X-ray films (A). The signals on the membranes were quantified by densitometry (B). +, positive control spots; -, negative control spots; 1, GM-CSF; 2, IL-10; 3, IL-1β; 4, IL-6; 5, NAP-2; 6, Rantes; 7, TNF-α; 8, GRO-α; 9, IL-8.

To further characterize the most prevalent lineages in Taiwan, we chose one Beijing ancient strain (M24) and one Beijing modern strain (W06) and analyzed their ability to induce immune responses by human MDM. After infection, the culture supernatants were collected to determine the amount of cytokines by using dedicated antibody arrays, which can detect 120 kinds of chemokines, growth factors and cytokines (Fig. 2A). Production of IL-1β, IL-6, IL-8, IL-10, GM-CSF, GRO-α (growth regulated oncogene-α), Rantes (regulated on activation, normal T cell expressed and secreted), and TNF-α by M24-infected macrophages was significantly higher than by W06-infected macrophages (Fig. 2B). Only NAP-2 (neutrophil-activating protein-2) was expressed at lower levels by M24-infected macrophages compared to W06-infected macrophages (Fig. 2B). The results indicate that the ancient Beijing lineage induces higher levels of pro-inflammatory cytokines than does the modern Beijing lineage.

### Poor survival of an EAI strain compared to Beijing strains in MDM

**Figure 3:**
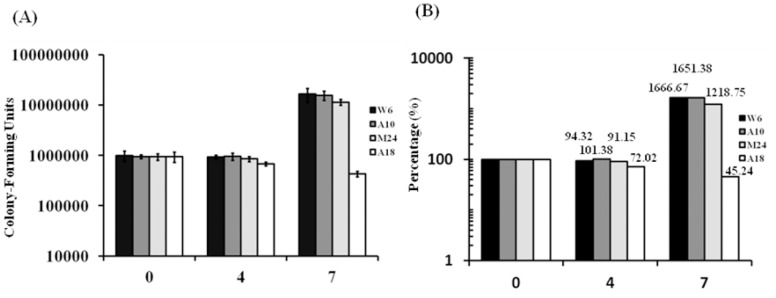
Poor survival of an EAI strain compared to Beijing strains in MDM. MDM were infected with different *M. tuberculosis* strains at a multiplicity of infection of 1:1 (bacterium to MDM ratio). The non-adhering bacteria were then eliminated with three washes with PBS. At specific time-points the bacterial uptake by MDM was determined by lysing a fraction of the cells with 0.1% saponin and counting the diluted cell lysates by plating. The results are presented as CFU (A) or percent survival relative to day 0 (B).

To compare survival ability among MTB groups, we used an MDM infection model to evaluate differences in intracellular growth. Four strains, W06 and A10 (modern Beijing), M24 (ancient Beijing) and A18 (EAI), were studied. There were no significant differences in bacterial uptake among the four stains at day 0 (Fig. 3). At day 4, the intracellular growth of W06 and M24 was slightly decreased to 94.32% and 91.15%, respectively, compared to day 0; that of A10 was essentially unchanged (101.38%); whereas that of A18 declined dramatically to 72.02%% compared to day 0 (Fig. 3). At day 7, the intracellular growth of three strains was significantly increased when compared with day 0 (Fig. 3). Especially noteworthy, the intracellular growth of A18 at day 7 was reduced to about 45.24% compared to day 0 (Fig 3). These results indicate that A18 (EAI strain) survives poorly in MDM compared to W06, A10 and M24 (Beijing strains).

It is known that many factors, including bacterial growth rate, are crucial for MTB survival inside human macrophages [16]. Therefore, we measured the growth rates of the above strains as described in Materials and Methods (Table 2); however, no significant differences among the three tested strains were found (one-way ANOVA, p = 0.099).

**Table 2:** Growth rate of different lineages of *Mycobacterium tuberculosis*.

Strain	Lineage	Doubling time^a^
H37Rv	Laboratory strain	21.61±2.01
W06	Modern (RD181□RD150▪ RD142▪)	22.77±3.06
M24	Ancient (RD181▪RD150▪ RD142▪)	19.53±3.08
A18	EAI	18.02±1.57

a No significant differences among the three tested strains (W06, M24 and A18) were found (one-way ANOVA, p = 0.099).

## Discussion

The most prevalent MTB strains in Taiwan belong to the Beijing lineage, followed by the EAI lineage [3], [4], [17]. The reasons for the high transmission of Beijing strains are not fully understood, but include a variety factors such as ineffective treatment of drug-resistant clones, selective pressure due to BCG vaccination [18], and human population migration and ethnicity [19], all of which have contributed to Beijing strain endemicity over Asia and elsewhere. Interestingly, the second most prevalent strains in Taiwan, EAI, have a higher frequency than Beijing strains in certain regions of the island, especially in southern and western Taiwan [4], [20]. Virulence variation in MTB strains remains controversial. However, MTB population structure is strongly clonal [21]; such clonal structure may be due to specific virulence characteristics [22].

Beijing lineages can be further divided into two major groups, ancient and modern, according to the presence or absence of IS6110 insertion or RD regions [14], [23], [24]. The prevalence of modern lineages (calculated by determining the insertion of an IS6110 element in the NTF chromosomal region) in Taiwan has been estimated to be 74% higher than for ancient strains [3]. A recent study by Portevin et al. found that infection with modern strains (according to the occurrence of the TbD1 genomic deletion) induced a significantly weaker immune response compared to ancient strains, as measured by levels of TNF-α, IFN-γ, IL-6, IL-10, IL12p40/p70, IL-15, MCP-1, MIP-1α, MIP-1β, VEGF, CXCL9 and Rantes [11]. Our results are compatible with previous findings [11], [25], in that the modern strains induced lower levels of TNF-α, IL-1β, IL-6, IL-8, IL-10, GRO-α, GM-CSF and Rantes, whereas NAP-2 was highly expressed when compared to ancient strains (Fig. 2 A and B). González-Cortés et al. reported that recombinant NAP-2 increased the activity of IFN-γ-activated MDM against *Legionella pneumophila* but not against MTB [26]. However, Khajoee et al. showed that MDM stimulated with CXCL-7 (NAP-2) had significantly enhanced antimicrobial activity against *Mycobacterium* [27]. The role of NAP-2 against MTB infection remains controversial.

It will be important to further explore the cytokine expression patterns of the most epidemic MTB strains, particularly the Beijing and EAI genotypes in Taiwan. Overall, EAI strains induced higher cytokine levels than Beijing strains (Fig. 1), and similar trends were also observed by Wang et al. [28]. The differences in pro-inflammatory cytokine expression patterns elicited by these two major groups of MTB may explain why Beijing strains are predominant in Taiwan.

However, heterogeneous cytokine expression was observed between the ancient and modern Beijing lineages (Fig. 1). Different RD regions in ancient and modern Beijing lineages may be the reason why the modern strains express lower levels of pro-inflammatory cytokines. To address this question, we subdivided the modern Beijing lineage into four groups, according to the presence or absence of RD150 or RD142, and two major groups were chosen for further assay. Our results show that, following infection of MDM, the modern Beijing sub-lineage possessing intact RD150 and RD142 induced very low levels of pro-inflammatory cytokines compared to the ancient Beijing lineage (with intact RD181, RD150 and RD142). Two genes, Rv2262c and Rv2263, are located in the RD181 region. Rv2262c is thought to be involved in lipid metabolism. Rocha-Ramirez et al. reported that cytokines can be negatively modified by lipids extracted from a virulent Beijing MTB genotype [29]. Furthermore, Rezwan et al. reported that the conceptual Rv2262 gene product has low but significant homology to *E. coli* apolipoprotein *N*-acyltransferase [30]. Inactivation of genes involved in lipoprotein synthesis attenuates the pathogenicity of MTB [30]. We hypothesize that RD181 contains genes which control the synthesis of cytokines and lipoproteins. Putative roles for genes in the RD181 region in regulating cytokine expression and lipid metabolism could be further investigated by transferring RD181 genes into modern Beijing strains and testing cytokine production, and by comparing the lipid profiles of ancient and modern Beijing sub-lineages. Aguliar et al. indicated that severe tissue damage and induction of lower levels of IFN-γ and nitric oxide synthase, and high but transient TNF-α expression, were associated with a high-virulence MTB phenotype, whereas mild tissue damage and progressive expression of IFN-γ and TNF-α were associated with a low-virulence MTB phenotype [31]. Based on our data, similar trends were observed in the most prevalent modern strains inducing low TNF-α and IFN-γ expression.

The expression level of TNF-α induced by MTB is correlated with the level of bacterial growth [16]. A rapid growth phenotype is associated with reduced TNF-α secretion, whereas robust TNF-α secretion inhibits mycobacterial replication [16]. Our results show that the EAI lineage, which induced a high level of TNF-α secretion, exhibited poor growth in MDM compared to the Beijing lineage (Fig. 3).

Other investigators have reported that Beijing strains grew faster in the lungs of mice and induced higher mortality than strains from the other lineages [32], [33]; therefore, we will further investigate the virulence of prevalent lineages of MTB in Taiwan using a mouse model to determine the survival rate and growth rate of each strain in mouse lung.

In summary, we have demonstrated that epidemic MTB strains exhibit different pro-inflammatory cytokine profiles, with modern Beijing strains that possess intact RD150 and RD142 regions inducing very low levels of pro-inflammatory cytokines when compared to ancient Beijing and EAI lineages. This is the first described cytokine profile of MTB that correlates with transmission dynamics in Taiwan.

## Supporting Information

Table S1 Clinical strains of *Mycobacterium tuberculosis* used in this study.
(DOC)

Table S2 Molecular cluster rates of ancient and modern Beijing strains.
(DOC)
