# Supplementary material for: The Pattern of Cytokine Production In Vitro Induced by Ancient and Modern Beijing Mycobacterium tuberculosis Strains
Source: PLoS One. 2014 Apr 11;9(4):e94296. doi: 10.1371/journal.pone.0094296 (PMC3984122; doi:10.1371/journal.pone.0094296)
Supplement: Table S1 — Clinical strains of Mycobacterium tuberculosis used in this study. (DOC) [file pone.0094296.s001.doc]

**Table S1. Clinical strains of *Mycobacterium tuberculosis* used in this study**

| **Lineage** | **Strains** | **Spoligotype** | **Average age^a^**  **(years)** | **Collection period** |
| --- | --- | --- | --- | --- |
| EAI | A18, B07, KVGH215, KVGH219,KVGH228,  KVGH248,KVGH272KVGH274,KVGH279  KVGH307 | 677777477413771 | 66.9 | 2003~2008 |
| **Beijing**  **Ancient lineage**  RD181◼RD150◼ RD142◼  **Modern lineage**  RD181🞏RD150◼ RD142◼  RD181🞏RD150 🞏RD142🞏 | A82,A88,B38,B55,B61,B65,C74,M24,M29,W26  A11,A35,C07,D05,D21,KVGH230,M09,W06,W60, W96  A10,A41,A80,B35,B62,B66,B96,C30,C51,D78 | 000000000003771 | 60.2  54.0  60.8  64.1  56.9 | 2003~2007 |

**^a^ The mean age of MTB-infected patients.**
