# Supplementary material for: The Pattern of Cytokine Production In Vitro Induced by Ancient and Modern Beijing Mycobacterium tuberculosis Strains
Source: PLoS One. 2014 Apr 11;9(4):e94296. doi: 10.1371/journal.pone.0094296 (PMC3984122; doi:10.1371/journal.pone.0094296)
Supplement: Table S2 — Molecular cluster rates of ancient and modern Beijing strains. (DOC) [file pone.0094296.s002.doc]

**Table S**2. Molecular cluster rates of ancient and modern Beijing strains

|  | No. of isolates in total | No. of isolates with a unique profile | No. of clusters | No of isolates in cluster | Strain-clustering rate (%) |
| --- | --- | --- | --- | --- | --- |
| Ancient | 31 | 17 | 5 | 14 | 29.03 |
| Modern | 307 | 157 | 45 | 150 | 34.20 |
| Total | 338 | 174 | 50 | 164 | 33.73 |
